# Comparative genomics of *Nocardia seriolae* reveals a conserved metabolic core and extensive accessory genome plasticity

**DOI:** 10.3389/fmicb.2026.1881314

**Published:** 2026-07-09

**Authors:** Kiran Kumar Eripogu, Payal Maharathi, Wen-Hsiung Li

**Affiliations:** 1 Biodiversity Research Center, Academia Sinica, Taipei, Taiwan; 2 Graduate Institute of Environmental Engineering (GIEE), National Taiwan University, Taipei, Taiwan; 3 Department of Ecology and Evolution, University of Chicago, Chicago, IL, United States

**Keywords:** accessory genome plasticity, biosynthetic gene clusters, fish nocardiosis, iron acquisition, *Nocardia seriolae*, open pangenome, persistence-associated adaptation

## Abstract

**Introduction:**

Fish nocardiosis is a chronic and economically significant bacterial disease in aquaculture, yet its genomic basis remains poorly resolved beyond single-species studies. It remains unclear whether fish-associated *Nocardia* share conserved persistence-associated features or exhibit lineage-specific genomic diversification.

**Materials and methods:**

We conducted a comparative genomic analysis of 22 *Nocardia* genomes, including 20 *N. seriolae* isolates and single representatives of *N. salmonicida* and *N. crassostreae*. Genome-wide analyses included phylogenomics, gene-content comparison, pangenome analysis, functional annotation, virulence-associated homolog screening, genomic island detection, and secondary biosynthetic gene cluster prediction.

**Results:**

The conserved genome core was enriched in central metabolism, lipid-associated cell envelope biogenesis, iron acquisition, and stress-response pathways. Virulence-associated homologs were dominated by persistence-associated and metabolic functions, whereas classical toxin systems were limited, although several transport- and secretion-associated homologs were detected, consistent with their potential contribution to host interaction and intracellular persistence. Phylogenomic and gene-content analyses revealed clear species-level divergence but limited host-associated structuring within *N. seriolae*. Pangenome analysis supported a robust open pangenome structure (*γ* = 0.386), with extensive accessory gene diversity enriched in regulatory functions, mobile genetic elements, and secondary metabolic pathways. Genomic islands were dominated by insertion-sequence-associated genes, recombinases, regulators, and hypothetical proteins, whereas prophage- and toxin-related signatures were rare. Secondary metabolite analysis revealed extensive biosynthetic diversity, with most biosynthetic gene clusters showing low similarity to characterized reference pathways. However, ectoine- and nocobactin-associated pathways were broadly conserved.

**Conclusion:**

These genome findings are consistent with a persistence-associated pathogenicity model in which fish-associated *Nocardia*, particularly *N. seriolae*, may depend more on metabolic resilience, stress adaptation, iron acquisition, and accessory genome plasticity than on classical toxin-mediated virulence. Collectively, the results highlight the importance of accessory genome diversification, iron acquisition, and stress adaptation in shaping host-associated lifestyles and provide a comparative genomic foundation for future functional investigations and aquaculture disease-management strategies.

## 1 Introduction

Aquaculture is the fastest-growing food production sector globally and is increasingly important for food security as capture fisheries approach ecological and economic limits. Intensification of aquaculture has also increased the burden of infectious diseases, particularly bacterial diseases, that reduce productivity, incur substantial economic losses, and threaten production sustainability (Bondad-Reantaso et al., 2005; Stentiford et al., 2017; FAO, 2024). Unlike acute viral epizootics that cause rapid mortality, many bacterial pathogens establish chronic infections that impair growth, suppress immunity, and increase susceptibility to secondary infections. This leads to prolonged production losses and increased management costs (Stentiford et al., 2012; FAO, 2012; Austin and Austin, 2007; Subasinghe, 2017).

Fish nocardiosis is a chronic granulomatous disease caused by members of the genus *Nocardia*, a group of aerobic, Gram-positive, partially acid-fast actinobacteria with lipid-rich cell envelopes (Shimahara et al., 2009; Liu et al., 2023). The disease affects both marine and freshwater fish species. It is characterized by systemic infection, chronic inflammation, tissue necrosis, and high cumulative mortality (Yasuike et al., 2017; Han et al., 2019). Disease control remains difficult because *Nocardia* spp. grow slowly, persist intracellularly, and are often misidentified with other granulomatous bacterial diseases (Han et al., 2019; Yetmar et al., 2023). Effective vaccines and targeted therapeutics are also limited (Nazareth et al., 2023; Zhou S. M. et al., 2024). Among fish-associated species, *N. seriolae* is the most extensively studied and widely reported, with outbreaks documented across Asia, Europe, and the Americas (Cao et al., 2023; Li et al., 2022; Zhang et al., 2024). In contrast, comparatively little genomic attention is available for *N. salmonicida* and *N. crassostreae* despite their association with fish or other aquatic hosts. The limited availability of genomic resources currently restricts broader comparative analyses of these species. This imbalance limits broader understanding of evolutionary diversity, metabolic variation, and persistence-associated traits across fish-associated *Nocardia*. Although fish nocardiosis is primarily an aquaculture problem, the persistence biology of *Nocardia* is relevant across host-associated contexts.

Comparative genomics provides a framework to investigate genomic conservation, accessory genome dynamics, metabolic adaptation, and secondary metabolism across related pathogens. Previous genomic studies of *N. seriolae* identified an open pangenome enriched in conserved metabolic and stress-associated functions (Cao et al., 2023; Li et al., 2022), whereas strain-level variation was largely restricted to regulatory and transport-related genes (Han et al., 2019; Xu et al., 2021; Eripogu et al., 2025). Pan-genome analyses of pathogenic *Nocardia* have similarly highlighted extensive accessory genome diversity and species-specific genomic signatures, further supporting the importance of comparative genomics for understanding adaptation and pathogenicity within the genus (Wang et al., 2025). Large-scale comparative genomic investigations have revealed extensive accessory genome diversification across the genus, with regulatory genes, mobile genetic elements, and biosynthetic gene clusters representing major contributors to genomic innovation and ecological adaptation (Eripogu et al., 2025). However, most available studies have focused on human-associated isolates or genus-wide datasets, leaving fish-associated *Nocardia* comparatively underexplored. Genome-scale analysis provides a useful framework for examining candidate persistence-associated pathways that may contribute to chronic *Nocardia* infections (Didelot et al., 2012; Yasuike et al., 2017; Xu et al., 2021). Existing pathological and immunological studies suggest that fish nocardiosis is driven primarily by long-term persistence rather than acute toxin-mediated host damage. Disease progression appears linked to intracellular survival, chronic inflammation, and sustained host colonization rather than classical exotoxin production. This pattern is consistent with nocardiosis observed in both fish and bivalve hosts (Paillard et al., 2004; Nazareth et al., 2024). Fish-associated *Nocardia* are therefore hypothesized to rely on conserved metabolic flexibility, lipid-rich cell-envelope functions, iron acquisition systems, and stress-response pathways that facilitate intracellular survival under host-imposed stress conditions. Immunoproteomic analysis further supports this model by showing that host immune responses predominantly target conserved housekeeping and stress-associated proteins rather than species-specific virulence effectors (Chen et al., 2019). Furthermore, experimental studies have demonstrated that disruption of persistence-associated pathways can significantly reduce virulence in *N. seriolae*. For example, disruption of the mycolic-acid biosynthesis gene *kasB* reduced host-cell invasion and attenuated virulence, supporting the importance of cell-envelope-associated persistence mechanisms rather than classical toxin production (Zhou S. M. et al., 2024). Likewise, pathological investigations have shown that fish nocardiosis is characterized by chronic granulomatous lesions and prolonged intracellular survival, consistent with persistence-driven disease progression (Nazareth et al., 2024; Zhou T. et al., 2024). *Nocardia* genomes also encode extensive and diverse repertoires of biosynthetic gene clusters (BGCs), involved in secondary metabolism (Männle et al., 2020; Eripogu et al., 2025). Although many secondary metabolites are associated with antimicrobial activity or microbial competition, their contribution to persistence, stress tolerance, and ecological adaptation in aquatic hosts remains unclear (Xu et al., 2022; Eripogu et al., 2025). Comparative analysis of BGC conservation and divergence may therefore provide insight into the ecological and evolutionary strategies of fish-associated *Nocardia*. Recent studies have increasingly suggested that *Nocardia* pathogenicity is governed by a combination of persistence-associated traits rather than classical toxin-mediated mechanisms. Comparative genomic, transcriptomic, and immunological investigations have highlighted the importance of iron acquisition, oxidative-stress defense, lipid metabolism, intracellular survival pathways, and secretion-associated functions in promoting long-term host colonization and disease progression (Liu et al., 2023; Nazareth et al., 2024; Zhou et al., 2024a, b). These findings underscore the need for comparative genomic analyses that evaluate virulence-associated functions within an evolutionary framework across multiple fish-associated *Nocardia* lineages.

Here, we performed comparative genomic analysis of 22 *Nocardia* genomes, comprising 20 *N. seriolae* isolates and single representatives of *N. salmonicida* and *N. crassostreae*. Because the dataset is dominated by *N. seriolae*, our analysis primarily focuses on intraspecies genomic structure, while the additional species provide a broader exploratory context. We used an integrated framework that includes phylogenomics, pangenome analysis, functional annotation, virulence-associated gene homolog profiling, and biosynthetic gene cluster identification. Specifically, we aimed to (i) characterize conserved metabolic and stress-associated genomic features, (ii) evaluate the conservation and distribution of virulence-associated gene homologs within and across lineages, and (iii) assess the diversity and organization of secondary metabolite gene clusters. Together, this analysis provides a genome-scale framework for understanding persistence-associated biology and accessory genome diversification in fish-associated *Nocardia*. This framework provides a basis for generating hypotheses for future functional and applied studies in aquaculture systems.

## 2 Materials and methods

### 2.1 Genome dataset and virulence-associated gene profiling

A total of 22 *Nocardia* genomes were analyzed, including 20 *N. seriolae* isolates and single representatives of *N. salmonicida* and *N. crassostreae* obtained from the NCBI database. At the time of data collection, only one publicly available genome was available for each of *N. salmonicida* and *N. crassostreae* in the NCBI database. These species were included because they represent aquatic-host-associated *Nocardia* discussed in the Introduction and provide a broader comparative context beyond *N. seriolae*. Manual inspection of BioSample metadata indicated that the broad-scale environmental context of *N. salmonicida* NBRC 13393 was reported as fish, whereas *N. crassostreae* NBRC 100342 was isolated from a diseased Pacific oyster. Although represented by single genomes, both species were retained to provide an exploratory interspecific context for comparison with *N. seriolae*.

Genome assembly quality was assessed using BUSCO v5.1.2 with the *Corynebacteriales_odb10 lineage* dataset (Manni et al., 2021). Protein-coding sequences were predicted from Prokka v1.14.6 (Seemann, 2014). Virulence-associated genes were identified by homology search against the Virulence Factor Database (VFDB) using the curated VFDB protein dataset as reference (Liu et al., 2022). Predicted protein sequences from all genomes were searched against VFDB using BLASTP. Hits were retained using an E-value threshold of 1e-10, ≥40% amino-acid identity, and ≥60% alignment coverage of the VFDB reference protein. Redundant VFDB assignments were collapsed at the query-protein level by retaining the highest score for each protein. Data processing and filtering were performed using custom Python scripts. Because VFDB includes both classical virulence determinants and general fitness-associated genes, VFDB hits were interpreted as virulence-associated homologs rather than experimentally validated virulence factors.

### 2.2 Antimicrobial resistance (AMR) gene analysis

AMR genes were screened using NCBI AMRFinderPlus (amrfinder v4.2.7), with the database version of 2026-03-24.1 under default parameters (Feldgarden et al., 2019). Genome assemblies were analyzed using nucleotide-based searches with the *--plus* option enabled. This option was used to include AMRFinderPlus-associated antimicrobial resistance and additional relevant gene categories where applicable. To further assess potential resistance-associated homologs, Comprehensive Antibiotic Resistance Database (CARD) protein homolog model sequences (Alcock et al., 2020) were searched against all genomes using tBLASTn (Camacho et al., 2009). Hits were retained only if they met stringent criteria of ≥80% amino acid identity, ≥70% query coverage, and ≥70% subject coverage.

### 2.3 Genomic islands (GIs) prediction

GIs were predicted only for the 20 *N. seriolae* genomes using IslandViewer 4 via the HTTP API framework (Bertelli et al., 2017). This species-restricted analysis was used to avoid overinterpreting genomic island variation from the singleton *N. salmonicida* and *N. crassostreae* representatives. Genome assemblies in GenBank format were submitted individually to the IslandViewer REST API. Draft genomes were reordered using the complete genome of *N. seriolae* EM150506 (RefSeq accession: NZ_CP017839.1) as the reference. Predictions were generated using the integrated IslandViewer framework, which combines sequence composition-based methods (SIGI-HMM and IslandPath-DIMOB) together with comparative genomic prediction (IslandPick) where applicable. Predicted GI-associated genes were summarized using custom Python scripts. Overlapping predictions were deduplicated prior to analysis.

### 2.4 Phylogenomic analysis

Single-copy orthologous genes were identified using OrthoFinder v2.5.4 with default parameters (Emms and Kelly, 2019). Single-copy orthologs were selected for phylogenomic reconstruction because they are expected to be vertically inherited and are less susceptible to complications arising from gene duplication, paralogy, and differential gene loss than multi-copy gene families. The use of conserved single-copy orthologs provides a robust framework for resolving evolutionary relationships among closely related bacterial strains and species while minimizing biases associated with accessory genome variation and horizontally acquired genes. Therefore, phylogenetic inference was based on the concatenated alignment of shared single-copy orthologs to capture the conserved evolutionary signal across the analyzed genomes. In this study, we used the set of 110 single-copy orthologous genes previously identified in Eripogu et al. (2025) and retained the orthologs shared among the 22 *Nocardia* genomes analyzed here. The corresponding protein sequences were concatenated and aligned using MAFFT v7.505 with default settings (Katoh and Standley, 2013). Phylogenetic reconstruction was performed using IQ-TREE v2.2.0.3 under a maximum-likelihood framework with automated model selection (Minh et al., 2020). The WAG+F + R2 model was selected as the best-fitting model. Branch support was assessed using 1,000 ultrafast bootstrap replicates and SH-like approximate likelihood ratio tests. Trees were visualized using iTOL v6 (Letunic and Bork, 2024).

### 2.5 Pangenome analysis and functional annotation

Pangenome analysis was performed using Roary (Page et al., 2015), with an 80% amino-acid identity threshold (i = 80). To assess the robustness of pangenome inference to dataset composition and clustering stringency, additional Roary analyses were performed using only the 20 *N. seriolae* genomes at i = 80 and also using the complete 22-genome dataset at different thresholds (85, 90, and 95%). Pangenome and core-genome accumulation curves were computed from the Roary presence-absence matrix (*gene_presence_absence. Rtab*). A total of 100 random genome permutations were used to minimize sampling bias. Pangenome openness was assessed using Heaps’ law (*P = k·N^γ*), where *P* represents the cumulative number of gene clusters, and *N* the number of genomes analyzed (Tettelin et al., 2005; Medini et al., 2005). Parameters k and γ were estimated by non-linear regression, with γ < 1 indicating an open pangenome. Gene presence-absence patterns were visualized alongside a Roary-compatible phylogenetic tree using custom Python scripts.

Functional annotations were performed using eggNOG 5.0 databases (Huerta-Cepas et al., 2019) and eggNOG-mapper v2 (Cantalapiedra et al., 2021). Orthologous groups were assigned using the DIAMOND mapping mode with the bacterial taxonomic scope. eggNOG-mapper outputs were used to infer functional categories, including COG classifications, KEGG ortholog assignments, and CAZy family annotations.

### 2.6 Biosynthetic gene cluster (BGC) identification and analysis

BGCs were identified using antiSMASH v6.0 (Blin et al., 2021) with relaxed detection strictness. Analysis was based on finalized antiSMASH regions and KnownClusterBlast similarity searches against MIBiG v3.0 references (Terlouw et al., 2023). Intermediate prediction layers and domain-level annotations were excluded to ensure consistent and conservative BGC classification across genomes.

## 3 Results

### 3.1 Conserved metabolic and cell envelope functions

The analyzed *Nocardia* genomes showed limited variation in genome size (7.5–8.37 Mbp) and GC content (67–68.3%). These features were broadly consistent across the analyzed isolates. Despite differences in assembly level, all genomes had BUSCO completeness scores above 96%, supporting robust comparative analysis (Supplementary Table S1). Across isolates, predicted gene counts ranged from approximately 6,300 to below 8,000 genes per genome, with *N. crassostreae* encoding 8,203 predicted genes. Protein-coding sequences (CDSs) closely mirrored total gene counts across all genomes. A high proportion of hypothetical proteins was observed in all genomes, accounting for ~50–55% of predicted CDSs (Supplementary Table S2).

Prokka revealed broad representation of core cellular and metabolic functions, including central carbon metabolism, energy generation, amino acid biosynthesis, lipid metabolism, and co-factor utilization. Housekeeping pathways, including glycolysis, the tricarboxylic acid cycle, oxidative phosphorylation, ribosomal proteins, and DNA replication and repair proteins, were consistently represented. Genes related to cell envelope biogenesis, stress response, transport, and regulation were also widely annotated. Genes encoding oxidative stress defense, including multiple copies of *catalase* and *superoxide dismutase* (SOD), were detected across all genomes. Enzymes potentially associated with host–microbe interaction, including phospholipase C and proteases, were also identified, although their functional roles require experimental validation. *N. seriolae* showed a relatively uniform repertoire, whereas *N. crassostreae* and *N. salmonicida* exhibited lower counts for some annotated categories (Supplementary Table S2).

### 3.2 Virulence-associated gene homologs

VFDB screening identified 336 to 399 virulence-associated homologs per genome. *N. seriolae* isolates showed highly conserved profiles (336–360 genes), with 241 homologs shared across all strains. These genes were dominated by functions related to amino acid biosynthesis and utilization, lipid metabolism, central carbon flux, and energy generation. Transport systems and regulatory proteins associated with nutrient acquisition and regulation were also consistently represented. Genes associated with stress tolerance, including redox homeostasis, nitrogen assimilation, and adaptation to nutrient limitation, were prevalent and frequently redundant (Supplementary Table S2). *N. salmonicida* and *N. crassostreae* encoded slightly larger counts (392 and 399 genes, respectively), with 88 homologs conserved across *N. seriolae*, *N. salmonicida*, and *N. crassostreae*. These genes were enriched for iron acquisition systems, including siderophore biosynthesis and transport components (*FbpABC, Fag, Dhb, Ent, Sit*), iron uptake and regulation genes (*feoB, fhuC, ideR*), and siderophore-associated exporters. Additional conserved functions included oxidative stress defense and redox maintenance, such as *MsrPQ* repair components, *hemerythrin-like proteins*, and *iron–sulfur cluster-associated* enzymes. Several toxin-antitoxin system components (*PemK/MazF*- and *AbiEii*-like) were also detected. No canonical exotoxin systems or well-characterized secretion-associated effector repertoires were identified. However, because these inferences are based on homology-based annotation, they should be interpreted as candidate persistence- and virulence-associated functions rather than experimentally validated virulence determinants. AMRFinderPlus detected no high-confidence acquired antimicrobial resistance determinants in the analyzed genomes. Consistently, the complementary CARD tBLASTn search produced no retained hits after applying stringent identity and bidirectional coverage filters.

### 3.3 Phylogenomic relationships and gene-content variation

**Figure 1:**
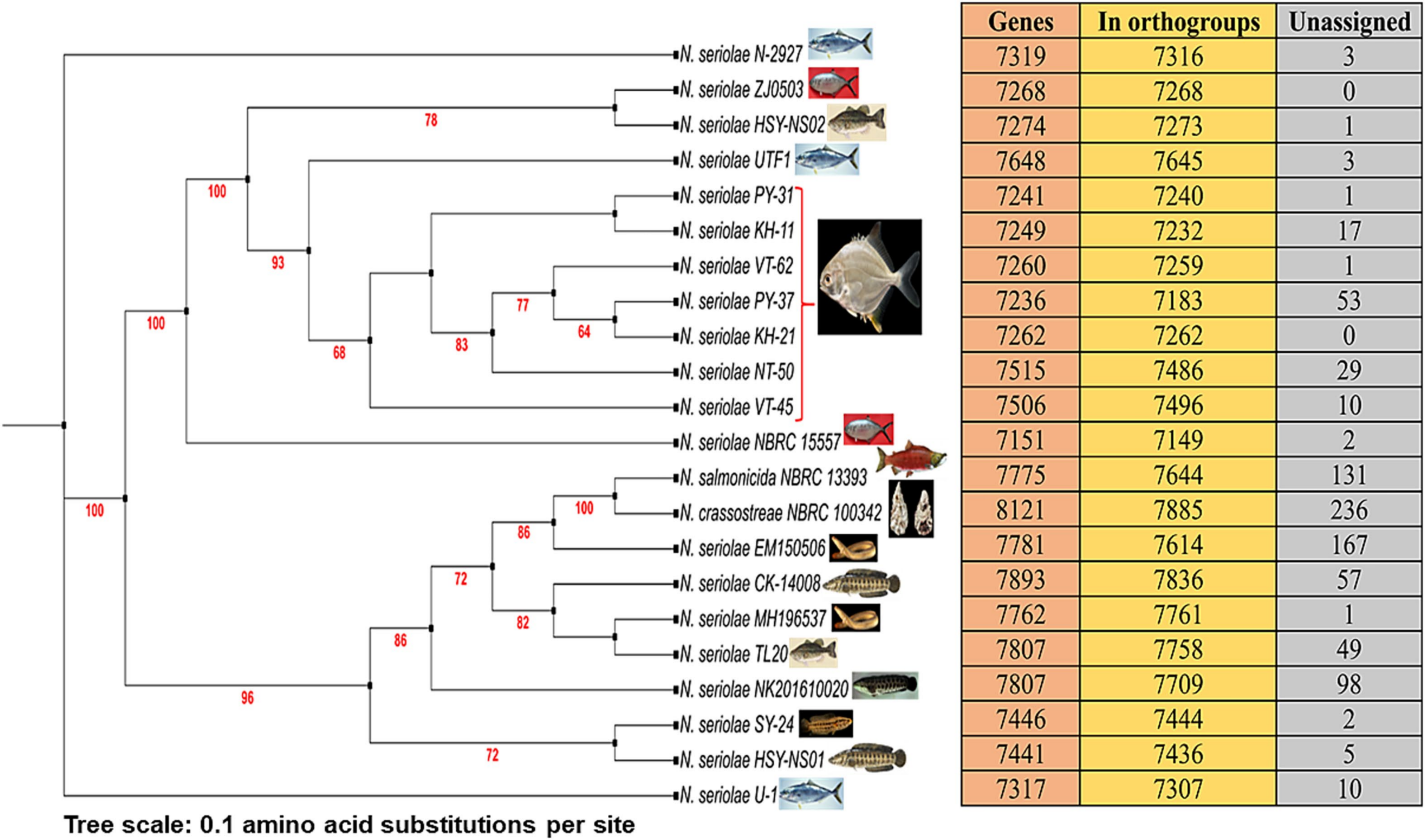
Maximum-likelihood phylogenomic tree based on concatenated 110 single-copy orthologs of 22 fish-associated *Nocardia* genomes was inferred using the WAG+F + R2 model for protein evolution with 1,000 ultrafast bootstrap replicates, with support values (percentage) indicated at internal nodes. Representative host fish images are shown alongside selected strains to indicate the host origin. The table on the right summarizes OrthoFinder-derived genome statistics for each strain, including the total genes, genes assigned to orthogroups, and unassigned genes.

Phylogenomic analysis based on concatenated single-copy orthologs resolved three well-supported species-level clades, corresponding to *N. seriolae*, *N. salmonicida*, and *N. crassostreae* (Figure 1). Deep internal nodes separating species were supported by high bootstrap values (≥96–100%). *N. salmonicida* and *N. crassostreae* each formed distinct, isolated branches, consistent with their species-level separation and distinct host/source associations (salmonid fish and oyster, respectively). Within *N. seriolae*, multiple well-supported subclades were observed, indicating substantial substructure. Several clusters partially correspond with host origin; for example, isolates associated with *Trachinotus* species are frequently grouped together. However, hosts such as *Seriola*, *Channa*, *Anguilla*, and *Micropterus* were often interspersed across the tree, and closely related strains were not always derived from the same host species (Figure 1).

Gene content analysis showed strong conservation among *N. seriolae* genomes. More than 99% of genes were assigned to orthogroups, and species-specific orthogroups were rare. This high degree of gene sharing corresponded to the tight clustering of *N. seriolae* genomes and reflected limited orthogroup variation within the species. In contrast, *N. salmonicida* and *N. crassostreae* contained larger numbers of unassigned genes (131 and 236, respectively) (Figure 1). *N. crassostreae* additionally encoded multiple species-specific orthogroups, consistent with its greater species-level divergence. Overall, gene content divergence was more pronounced at the species level than among *N. seriolae* strains.

### 3.4 Pangenome structure and accessory gene dynamics

**Figure 2:**
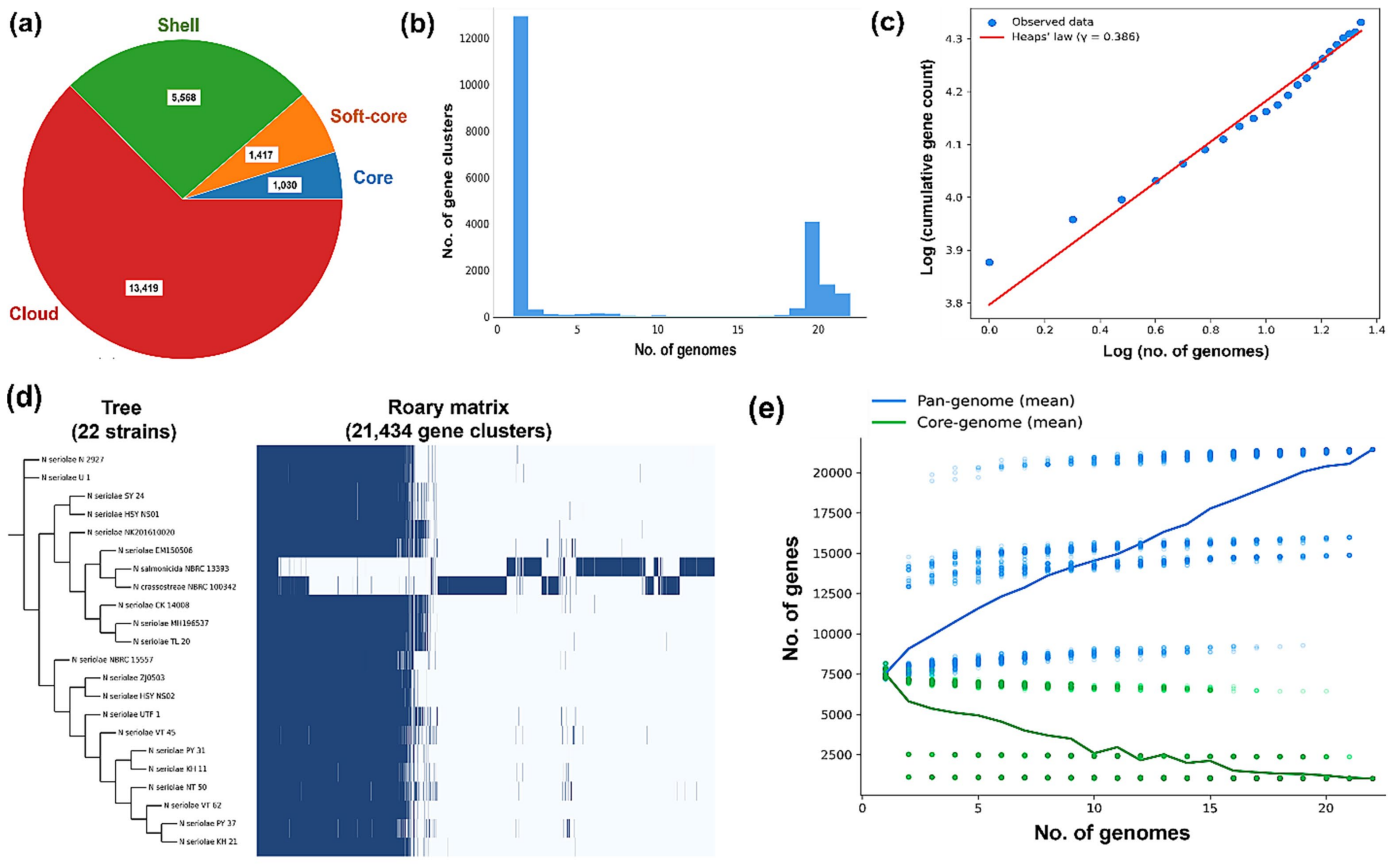
Roary-based pangenome analysis of 22 fish-associated *Nocardia* genomes. **(a)** Distribution of gene clusters across pangenome compartments, showing the relative contributions of the core, soft-core, shell, and cloud genomes. **(b)** Frequency distribution of gene clusters across genomes. **(c)** Heaps’ law fitting of pangenome expansion, demonstrating a power-law relationship (*γ* < 1) between the number of genomes sampled and the total number of gene clusters, consistent with an open pangenome. **(d)** Presence-absence heatmap of Roary gene clusters across genomes, ordered according to the corresponding phylogenomic tree shown on the left, highlighting conserved core gene blocks and strain-specific accessory regions. **(e)** Pangenome and core-genome accumulation curves showing continuous growth of the pangenome and a gradual reduction of the core genome as additional genomes are incorporated.

Roary at 80% amino acid identity threshold for 22 genomes identified a total of 21,434 gene clusters. The core genome, defined as gene clusters present in all genomes, comprised 1,030 clusters, representing the most conserved genetic component shared across strains (Figure 2a; Supplementary Table S3A). Functional annotation revealed that the core genome was enriched in central metabolism (including glycolysis and TCA cycle enzymes), DNA replication and repair (DNA polymerase subunits, helicases, recombination proteins), transcription and translation machinery (RNA polymerase subunits, ribosomal proteins), and basic cell-envelope biogenesis. Stress-associated housekeeping systems, including chaperones and redox homeostasis proteins, were also conserved (Supplementary Tables S4, S5). The soft-core genome included 1,417 gene clusters; core and soft-core genes together accounted for 2,447 gene clusters (11.4%) of the total pangenome. The soft-core genome was enriched for membrane-associated proteins, transport systems (ABC-type, MFS transporters), transcriptional regulators, and lipid metabolism enzymes. Many soft-core genes reflected genes conserved across most *N. seriolae* genomes but absent from one or both singleton non-seriolae genomes (Supplementary Tables S3A, S4, S5).

The accessory genome represented the dominant component of the pangenome, comprising 18,987 gene clusters (88.6%) (Figure 2a). This accessory component was subdivided into a shell genome of 5,568 clusters (26.0%), representing genes shared by subsets of strains, and a cloud genome of 13,419 clusters (62.6%), consisting of genes present in only a few genomes. Shell genes were functionally diverse and included transporters, transcriptional regulators, secretion-associated proteins, mobile element-linked genes, and enzymes involved in secondary metabolism and lipid modification (Supplementary Tables S3A, S4, S5). In contrast, the cloud genome was dominated by hypothetical proteins, transposases, integrases, phage-associated proteins, and poorly characterized enzymes (Supplementary Tables S4, S5).

Gene frequency distributions showed strong polarization between highly conserved and low-frequency genes (Figure 2b). To quantify pangenome expansion, gene accumulation was modeled using Heaps’ law (Figure 2c). The fitted model yielded *γ* = 0.386, consistent with an open pangenome structure. The gene presence-absence matrix mapped onto the phylogenetic tree provides a genome-wide view of conserved and variable gene content (Figure 2d). *N. salmonicida* and *N. crassostreae* lacked several gene clusters conserved across most *N. seriolae* genomes. Outside the conserved core, shell, and cloud genes displayed fragmented and discontinuous presence-absence patterns across the phylogeny, including among closely related *N. seriolae* lineages. These patterns indicate that most genome variability was concentrated within the accessory genome. The pangenome and core genome accumulation curves illustrate contrasting trends in gene conservation and variability (Figure 2e). The mean pangenome size increased steadily with sequential genome addition and did not reach saturation, whereas the mean core genome size decreased rapidly during early genome additions before approaching a stable value.

Supplementary pangenome analysis restricted to the 20 *N. seriolae* genomes at the same 80% identity threshold showed that the pangenome remained open, with approximately 30% of the gene clusters belonging to the accessory genome. This indicates that substantial accessory gene diversity was retained within *N. seriolae* and was not solely attributable to inclusion of *N. salmonicida* and *N. crassostreae* (Supplementary Table S3B). Additionally, 22-genome pangenome analysis performed at 85, 90, and 95% amino acid identity thresholds showed the expected effect of increasing clustering stringency, including progressive reductions in core genome size and expansion of shell and cloud gene partitions (Supplementary Table S3A). Despite these shifts in ortholog clustering, the overall inference of extensive accessory genome diversity and an open pangenome structure remained consistent across thresholds.

### 3.5 Functional annotation and carbohydrate-active enzymes

**Figure 3:**
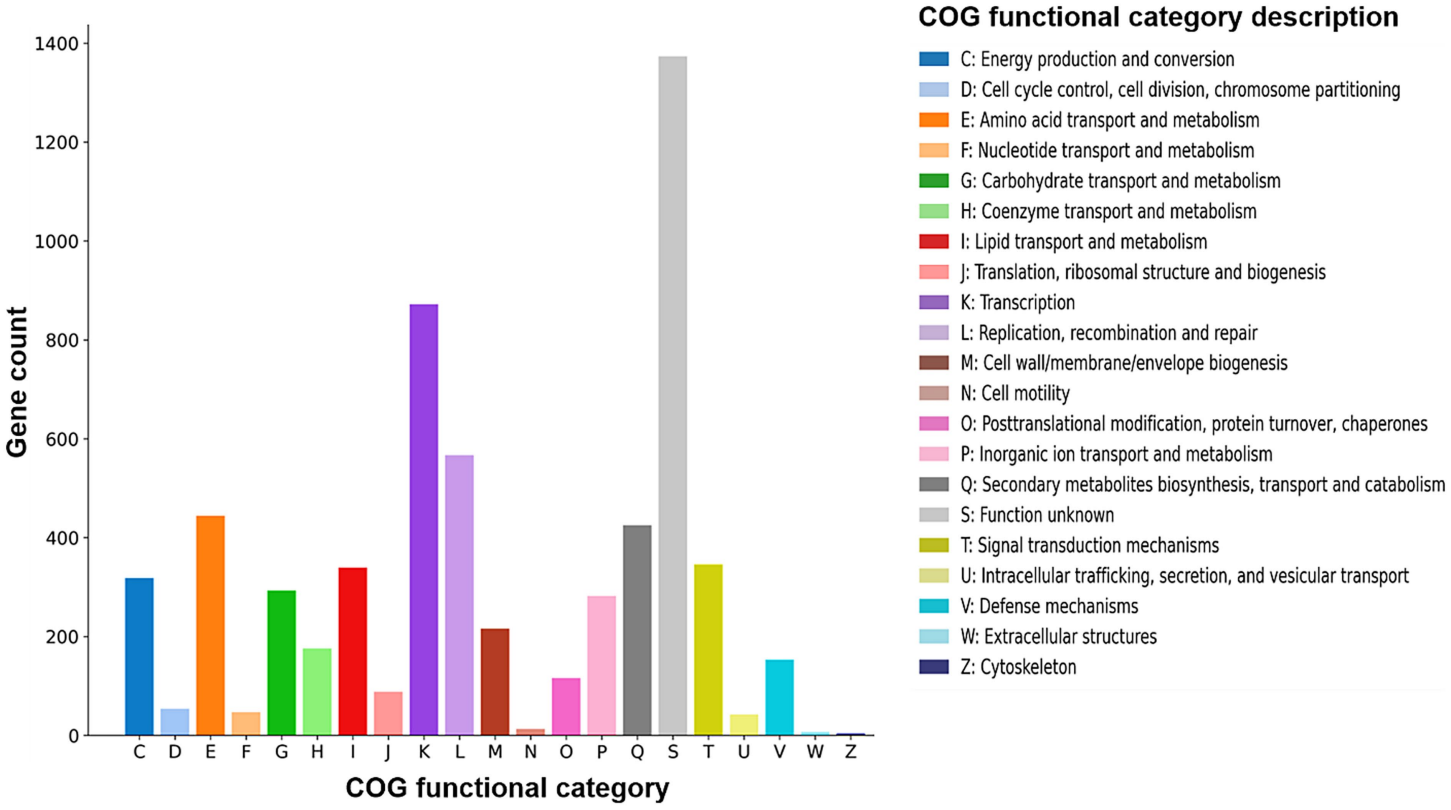
COG functional category distribution across *Nocardia* genomes. Each bar represents the total number of genes mapped to a given COG category.

COG-based functional classification revealed a highly uneven functional distribution (Figure 3; Supplementary Table S5). The largest fraction of gene clusters was assigned to function unknown (S). Among annotated categories, transcription (K) and replication, recombination, and repair (L) were most abundant, followed by amino acid (E) and lipid transport and metabolism (I), secondary metabolism (Q), and signal transduction (T). Gene clusters related to cell motility (N), intracellular trafficking (U), extracellular structures (W), and cytoskeleton (Z) were rare (Figure 3; Supplementary Table S5). KEGG orthologs associated with core metabolic pathways, including glycolysis and the tricarboxylic acid cycle, were detected across all genomes (Supplementary Table S5). Genes involved in amino acid biosynthesis and metabolism were also widely conserved. Two-component regulatory systems were present across genomes with variation in gene counts but not in functional categories (Supplementary Table S5).

**Figure 4:**
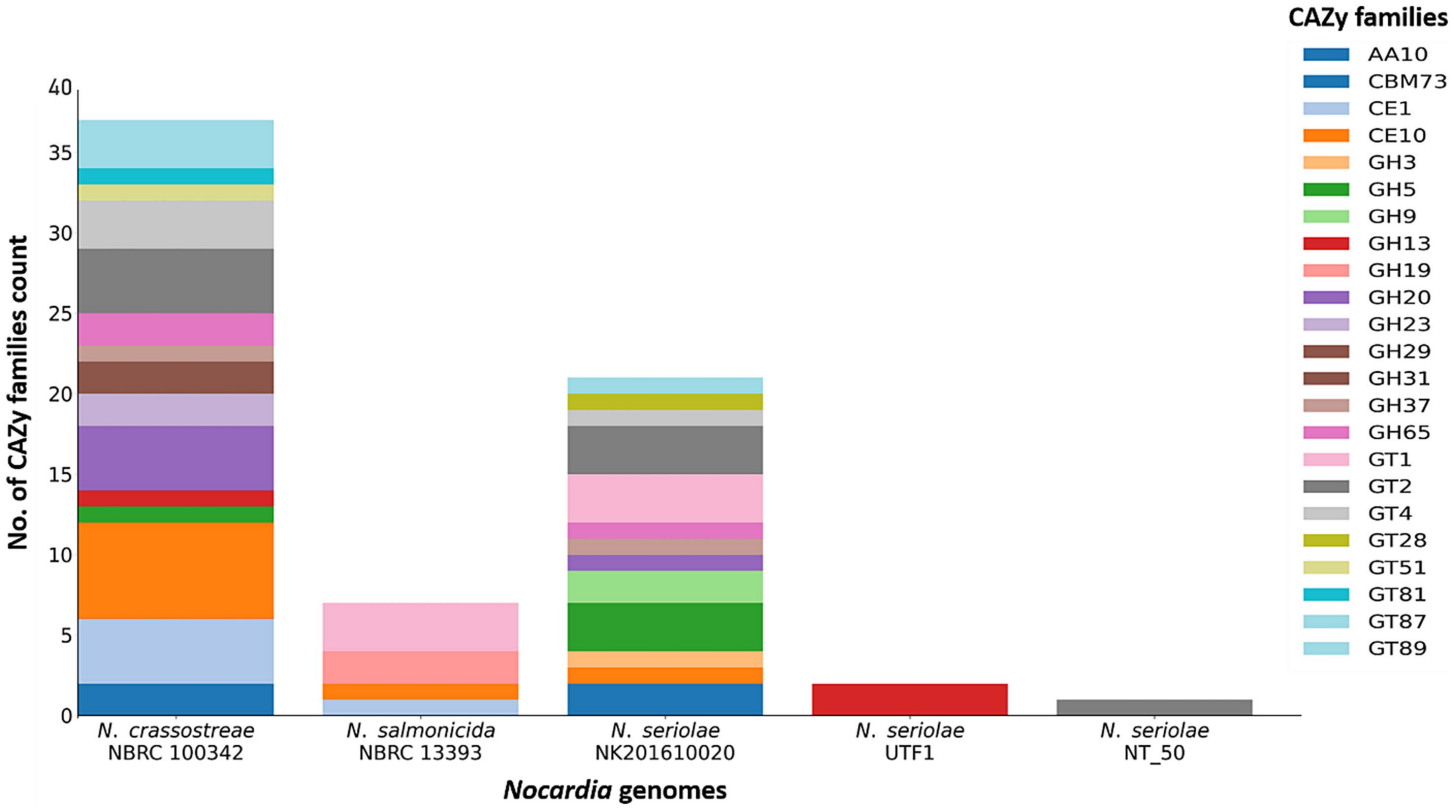
CAZy family composition across *Nocardia* genomes. The stacked bar chart showing the distribution of CAZy families across representative *Nocardia* genomes. Each bar corresponds to one genome, and colored segments indicate individual CAZy families.

The CAZy landscape in *Nocardia* was sparse, with low diversity and low copy number of carbohydrate-active enzymes (Figure 4; Supplementary Table S5). Glycosyltransferases (GTs) represent the most prominent CAZy class and include *GT1*, *GT2*, *GT4*, *GT28*, *GT51*, *GT87*, and *GT89*. These families were mainly associated with cell wall and envelope biosynthesis. Glycoside hydrolases (*GH*s) families, including *GH5*, *GH9, GH13, GH19, GH20, GH23, GH29, GH37, and GH65,* were detected at low copy number. The *N. crassostreae* genome displayed the broadest CAZy representation. It encoded multiple carbohydrate esterases (CEs) (*CE1*, *CE10*), repeated *GH20*, *GH23*, and *GH65* entries. It also encoded enzymes associated with cellulose modification, including *GH5* and an auxiliary activity (AA) enzyme, including lytic polysaccharide monooxygenase (*AA10*) with a carbohydrate-binding module (*CBM73*) (Figure 4; Supplementary Table S5). While this indicates a modestly expanded capacity for carbohydrate and polymer modification, the repertoire remains limited and substantially lower than that reported for dedicated polysaccharide-degrading bacteria.

### 3.6 Genomic islands composition and mobile-element-associated variability

Predicted genomic islands (GIs) varied substantially among *N. seriolae* genomes, ranging from 12 to 108 regions per genome, encompassing 486–2,636 predicted GI-associated genes (Supplementary Table S6A). Total genomic island content ranged from approximately 0.25–1.21 Mbp per genome. We observed a considerable heterogeneity in genomic island burden among *N. seriolae* genomes. Mean genomic island lengths ranged from 9–11 kb, although several genomes with fewer predicted islands exhibited larger average island sizes (>18 kb). Predicted genomic islands were dominated by hypothetical proteins and mobile-element-associated functions. After removal of duplicated annotations arising from overlapping island predictions, the most abundant identifiable functional category consisted of transposases, with 253 unique transposase-associated genes (Supplementary Table S6B). Multiple insertion-sequence families were represented, including *IS3*-, *IS4*-, *IS5*-, *IS30*-, *IS256*-, *IS701*-, and *IS1380*-associated transposases (Supplementary Table S6C). Recombinases (71 genes), transcriptional regulators (140 genes), and transporter-associated proteins (97 genes) were also frequently detected within predicted islands (Supplementary Table S6B).

In contrast, prophage-associated signatures were comparatively rare. Only seven phage-associated annotations and two integrase-associated genes were identified after deduplication. Most phage-associated annotations corresponded to phage shock protein A (*PspA*), a membrane stress response protein, while only two regions encoded putative prophage *phiRv2* integrases. Similarly, toxin-associated annotations were also uncommon and limited mainly to endoribonuclease *MazF3*, a component of toxin-antitoxin systems (Supplementary Table S6D). No obvious classical exotoxin, pore-forming toxin, or secretion-system-associated virulence annotations were detected among the predicted genomic islands. Collectively, these patterns indicate that predicted genomic islands in *N. seriolae* are dominated by insertion-sequence-associated and recombination-related functions. In contrast, prophage- and toxin-related signatures were comparatively limited.

### 3.7 Secondary metabolite diversity and distribution

A total of 1,219 BGCs were identified, of which 656 (53.8%) showed detectable similarity to MIBiG entries, while 563 (46.2%) lacked similarity with the MIBiG reference cluster. On average, each genome contained 55.4 ± 16.5 BGCs. NRPS-associated clusters represented the dominant class (35%), followed by hybrid BGCs (21%) and terpene-related clusters (15%) (Supplementary Table S7). Across *N. seriolae* genomes, multiple hybrid BGC classes were detected, including NRPS-ectoine-NAPAA, NRPS-terpene, NRPS-like, RiPP-like, T1PKS, ectoine, and hglE-KS-associated clusters. *N. salmonicida* genome encoded a reduced number of NRPS clusters compared with most *N. seriolae* strains. The *N. crassostreae* genome comprised NRPS-NRPS-like, NRPS-like-T1PKS, arylpolyene-associated, and T3PKS-associated clusters (Supplementary Table S7).

Most BGCs showed only partial similarity to characterized MIBiG reference clusters, suggesting a substantial divergence from characterized biosynthetic pathways. The ectoine-associated cluster represented a notable exception, showing 100% KnownClusterBlast similarity to the reference ectoine cluster (Supplementary Table S7). Genes encoding *ectA, ectB, ectC*, and, in some cases, *ectD* were consistently identified across genomes, supporting conservation of osmoprotectant biosynthesis and stress-tolerance functions. Genes associated with siderophore biosynthesis, ferric transport, and iron-responsive regulators, NRPS-associated enzymes, and siderophore transport systems were widespread across the analyzed genomes.

Siderophore-associated BGCs displayed a clear lineage-structured distribution. Nocobactin-like clusters were conserved across fish-associated *Nocardia* genomes and showed high similarity (75%) to the nocobactin NA reference clusters BGC0001027, supporting a conserved role in iron acquisition (Supplementary Table S8). Additional siderophore-associated systems, including *mycobactin* and *streptobactin*-like clusters, were also widely distributed, although these exhibited lower similarity to characterized pathways (~30% similarity to BGC0001021 and 11% similarity to BGC0000368, respectively). In contrast, *heterobactin*-associated clusters were detected only in *N. salmonicida* NBRC 13393 and *N. crassostreae* NBRC 100342, where two NRPS-associated BGCs showed 72% similarity to the *heterobactin A*/*S2* reference cluster BGC0000371 (Supplementary Table S8). *Scabichelin*-associated clusters, represented by mixed NRPS-like/NRPS/hglE-KS systems, showed approximately 30% similarity to BGC0000423. *Foxicin*-associated hybrid NRPS-T1PKS clusters showed only 4% similarity to BGC0001598. Both cluster types were restricted to *N. crassostreae* NBRC 100342. *Gobichelin*-associated clusters were identified only in *N. seriolae* HSY-NS01 as NRPS core BGCs showing low similarity (11%) to BGC0000366. Together, these patterns indicate that nocobactin-type systems are broadly conserved. Several siderophore-associated pathways exhibit lineage- or strain-specific distributions and substantial divergence from characterized reference clusters.

Antibiotic- and terpene-associated BGCs exhibited substantial divergence from characterized MIBiG pathways, with most clusters showing only weak similarity to known reference systems. *Echinomycin*-, *yatakemycin*-, and *zorbamycin*-like clusters were broadly distributed among *N. seriolae* genomes (Supplementary Table S8). However, *N. salmonicida* and *N. crassostreae* encoded more lineage-restricted antibiotic-associated pathways, including *salinomycin*-, *malacidin*-, *borrelidin*-, and *nanchangmycin*-like clusters. The generally low sequence similarity observed across these BGCs suggests that many represent evolutionarily diverged or potentially uncharacterized secondary metabolite systems rather than canonical biosynthetic pathways. Terpene-associated clusters showed stronger lineage specificity. A *geosmin* cluster was detected only in *N. salmonicida,* whereas a *2-methylisoborneol*-associated (2-MIB) cluster was detected only in *N. crassostreae*. Both showed 100% KnownClusterBlast similarity to their corresponding pathways, BGC0000661 and BGC0000657. Collectively, these patterns indicate that secondary metabolism in fish-associated *Nocardia* is shaped by lineage-specific diversification and retention of distinct biosynthetic capacities.

## 4 Discussion

The analyzed *Nocardia* genomes, dominated by *N. seriolae*, were characterized by highly conserved metabolic and stress-associated functions combined with extensive accessory genome diversity. Conserved pathways involved central metabolism, lipid metabolism, cell-envelope biogenesis, iron acquisition, and oxidative stress defense, whereas most variability occurred in regulatory genes, secondary metabolism-associated loci, and hypothetical proteins. This conserved functional backbone is consistent with previous evidence that nocardiosis may be primarily persistence-associated disease rather than an acute toxin-mediated infection (Han et al., 2019; Nazareth et al., 2024). Although classical exotoxin systems were not prominent, secretion- and transport-associated functions identified in the genomes may contribute to intracellular survival, nutrient acquisition, and host interaction, as reported previously for pathogenic *Nocardia* and related actinobacteria (Liu et al., 2023; Zhou et al., 2024). Consistent with this persistence-associated model, virulence-associated homologs were dominated by iron acquisition systems, redox maintenance pathways, nutrient acquisition functions, and toxin-antitoxin modules. Iron acquisition systems, particularly nocobactin-associated pathways, were highly conserved and may play central roles during host colonization under iron-limited conditions. Similar persistence-associated functions have been reported in other actinobacterial pathogens (Xu et al., 2021; Eripogu et al., 2025). The persistence-associated model proposed here is also consistent with previous genomic and pathological investigations of fish nocardiosis. Analysis of the complete genome of *N. seriolae* UTF1 revealed a large repertoire of genes associated with lipid metabolism, stress adaptation, and intracellular survival, suggesting that long-term persistence within host tissues is a central component of pathogenesis rather than acute toxin-mediated damage (Yasuike et al., 2017). Similarly, studies have emphasized the importance of the lipid-rich mycolic-acid-containing cell envelope, which contributes to resistance against host immune responses, antimicrobial exposure, and environmental stress (Nazareth et al., 2023; Liu et al., 2023). The predominance of iron-acquisition systems, oxidative-stress-response genes, and persistence-associated homologs observed in the present study further supports the hypothesis that successful host colonization depends on physiological resilience and adaptation to nutrient-limited intracellular environments. These observations suggest that persistence-associated mechanisms are evolutionarily conserved across fish-associated *Nocardia* and likely represent fundamental determinants of pathogenic success. Similar patterns have also been reported for *N. cyriacigeorgica*, where comparative genomic analysis identified conserved virulence-associated genes involved in intracellular survival, iron acquisition, and host interaction (Yang et al., 2025). Recent studies of both aquatic- and human-associated *Nocardia* further support the importance of persistence-associated traits in pathogenesis (Nazareth et al., 2024; Zhou et al., 2024b). Collectively, these findings indicate that stress adaptation, iron acquisition, and intracellular survival are conserved determinants of pathogenicity across the genus rather than lineage-specific strategies. Therefore, the genomic signatures identified in this study should be interpreted as being consistent with a persistence-associated pathogenicity model previously supported by experimental, pathological, and immunological studies of fish nocardiosis, rather than as direct functional evidence of persistence mechanisms.

At the evolutionary level, phylogenomic analysis based on conserved single-copy orthologs resolved clear species-level clades but showed limited evidence for strong host-associated clustering within *N. seriolae*. Because single-copy orthologs are generally less affected by gene duplication, gene gain-and-loss dynamics, and horizontal gene transfer than accessory genes, they are widely regarded as reliable markers for bacterial evolutionary reconstruction. The resulting topology, therefore, likely reflects the underlying vertical evolutionary history of the analyzed lineages rather than recent accessory genome acquisition events. Several closely related isolates originated from different fish hosts, further indicating that host species alone may not be the primary determinant of genomic structure within *N. seriolae*. The lack of strict host-associated clustering suggests that *N. seriolae* may behave as a relatively generalist aquatic pathogen capable of infecting multiple fish hosts. However, the present database was not sufficiently balanced across host species to perform robust host-associated accessory gene enrichment analysis. Therefore, host adaptation should be interpreted cautiously, and future studies should test accessory gene enrichment, host-origin association, and population structure using larger host-balanced datasets. This pattern may reflect environmental transmission through shared aquaculture water systems, movement of infected fish stocks, or circulation of common environmental reservoirs within aquaculture facilities. Similar observations have been reported for other aquatic bacterial pathogens, where geographic proximity and farming practices exert stronger influences on population structure than host species identity. The available data suggest greater gene-content divergence between the three represented species than among *N. seriolae* strains; however, broader species sampling will be required to confirm this pattern. This evolutionary pattern was further supported by pangenome analysis, which demonstrated a robust open pangenome structure with extensive accessory genome diversity, even within *N. seriolae* alone. Accessory regions were enriched in hypothetical proteins, transposases, recombinases, and regulatory genes, suggesting that genome plasticity is driven primarily by insertion-sequence-mediated diversification and accessory-region remodeling. In contrast, prophage-associated signatures and predicted islands carrying classical virulence-associated annotations were comparatively uncommon. Despite extensive accessory genome diversity, CAZy analysis indicated limited carbohydrate specialization. The low abundance of glycoside hydrolases and predominance of glycosyltransferases suggest that carbohydrate-associated functions are directed mainly toward cell-envelope maintenance rather than environmental polysaccharide degradation. The open pangenome structure observed in this study is consistent with large-scale comparative genomic investigations of *Nocardia*. Recent pan-genome studies have further demonstrated that species-specific core genes and accessory genome variation contribute substantially to diversification and adaptation across pathogenic *Nocardia* lineages (Wang et al., 2025). A comparative analysis of *Nocardia* genomes revealed extensive accessory genome expansion, high genomic fluidity, and enrichment of regulatory genes, mobile genetic elements, and biosynthetic loci outside the conserved core genome (Eripogu et al., 2025). These findings support the view that accessory genome diversification is a major driver of adaptation within the genus. The predominance of transposases, recombinases, regulators, and hypothetical proteins identified here is therefore consistent with broader evolutionary trends across *Nocardia*. Such genome plasticity may facilitate adaptation to diverse aquatic environments, host species, and ecological niches while maintaining a conserved metabolic core essential for survival and growth.

A similar pattern of conservation and diversification was observed in secondary metabolism. BGC analysis identified extensive biosynthetic diversity dominated by NRPS-derived systems, although most clusters showed only partial similarity to characterized reference pathways, indicating substantial evolutionary divergence and potentially novel biosynthetic capacity. Ectoine biosynthesis was the only fully conserved pathway, supporting an important role in osmoprotection and stress tolerance. Conserved siderophore-associated systems further emphasize the likely importance of iron acquisition during persistence in aquatic hosts. The broad conservation of nocobactin-like systems may reflect selective pressure for iron acquisition in host-associated and iron-limited aquatic environments. In contrast, lineage- and strain-specific siderophore-associated clusters may contribute to ecological diversification, microbial competition, or niche-specific survival, although their functional roles require metabolomic and experimental validation. The extensive diversity of BGCs identified in fish-associated *Nocardia* also agrees with previous comparative studies demonstrating that secondary metabolism represents one of the most dynamic components of the genus. Comparative genomic and metabolomic analyses have shown that *Nocardia* species possess diverse repertoires of NRPS, PKS, terpene, and hybrid biosynthetic pathways, many of which exhibit limited similarity to experimentally characterized metabolites (Männle et al., 2020). Furthermore, genome-scale analysis revealed that approximately one-third of *Nocardia* BGCs lack close similarity to known MIBiG reference BGCs, highlighting the substantial unexplored biosynthetic potential of the genus (Eripogu et al., 2025). The predominance of lineage-specific and low-similarity BGCs observed in the present study supports the hypothesis that secondary metabolism contributes to ecological adaptation, microbial competition, and niche specialization. Furthermore, the conservation of ectoine and siderophore-associated pathways suggests that some biosynthetic functions may be maintained by strong selective pressures associated with osmotic stress tolerance and iron acquisition in aquatic environments.

In this study, no high-confidence acquired antimicrobial resistance determinants were detected despite AMRFinderPlus screening and complementary CARD-based homology searches. This observation is broadly consistent with previous genomic studies of *N. seriolae*, which reported limited evidence of horizontally acquired resistance genes despite the recognized difficulty of antimicrobial treatment and the occurrence of variable susceptibility profiles among isolates (Han et al., 2019; Liu et al., 2023). The absence of detectable acquired AMR determinants does not exclude the presence of intrinsic resistance mechanisms, regulatory adaptations, physiological tolerance, or resistance determinants that remain uncharacterized in current databases. In particular, the lipid-rich cell envelope of *Nocardia*, together with potential efflux systems and persistence-associated physiological states, may contribute substantially to reduced antimicrobial susceptibility and long-term survival under treatment pressure (Liu et al., 2023; Yetmar et al., 2023). Similar discrepancies between genomic AMR predictions and observed phenotypic resistance have been reported in *Nocardia* and other actinomycetes. Comparative analyses of clinical *Nocardia* isolates have shown that antimicrobial susceptibility patterns are not always explained by currently recognized resistance determinants. These suggests important contributions from intrinsic resistance mechanisms, regulatory pathways, cell-envelope properties, and yet-uncharacterized resistance genes (Brown-Elliott et al., 2006; Yetmar et al., 2023). Similar genotype–phenotype discordance has also been documented in related mycolic-acid-containing actinobacteria, where resistance phenotypes may arise from complex physiological and regulatory mechanisms that are incompletely represented in current AMR databases. Therefore, phenotypic antimicrobial susceptibility testing will be necessary to determine the clinical and aquaculture relevance of resistance in fish-associated *Nocardia*.

Several limitations of this study should be acknowledged. Virulence-associated genes and biosynthetic gene clusters were inferred computationally and therefore require experimental validation. Consequently, the persistence-associated pathogenicity model proposed here should be considered a genome-informed hypothesis rather than a functionally demonstrated mechanism. In addition, only a single genome was available for each of *N. salmonicida* and *N. crassostreae* at the time of analysis, limiting assessment of intraspecific genomic diversity and preventing robust species-level comparative analyses. Consequently, observations involving these species should be interpreted as exploratory. Accordingly, the persistence-associated pathogenicity model proposed here should be considered a genome-informed hypothesis rather than a functionally demonstrated mechanism. Future studies integrating functional genomics, metabolomics, antimicrobial susceptibility testing, and infection models will be necessary to validate the biological significance of the genomic patterns identified here.

## 5 Conclusion

This study provides a comparative genomic framework for understanding persistence-associated biology in *N. seriolae* and related aquatic *Nocardia*. Despite extensive accessory genome diversity, the analyzed genomes shared a highly conserved metabolic core centered on central metabolism, lipid-associated cell-envelope functions, iron acquisition, and stress adaptation. In contrast, genomic variability was concentrated within accessory regions enriched in mobile genetic elements, regulatory functions, and secondary metabolism-associated loci, highlighting genome plasticity as an important contributor to ecological adaptation and diversification. These findings are consistent with a genome-informed persistence-associated pathogenicity model in which fish-associated *Nocardia* may rely more on physiological resilience and long-term host colonization than on classical toxin-mediated virulence. Secondary metabolite analysis revealed extensive biosynthetic diversity dominated by NRPS-associated systems, with many clusters showing only partial similarity to characterized pathways. Conserved ectoine- and siderophore-associated systems suggest candidate roles in osmotic adaptation and iron acquisition during host persistence. However, lineage-restricted BGCs suggest substantial unexplored metabolic diversity.

Overall, these results refine our current understanding of fish-associated *Nocardia* biology and evolution and are consistent with a persistence-associated model of pathogenicity. However, this model should be considered a genome-informed hypothesis that requires experimental validation through functional, phenotypic, and infection-based studies. Future studies integrating functional genomics, metabolomics, infection models, and antimicrobial susceptibility testing will be essential to validate the biological significance of these genomic features. Such studies will also be crucial for improving diagnostic surveillance, therapeutic strategies, and long-term disease management in aquaculture systems.

## Data Availability

The original contributions presented in the study are included in the article/Supplementary material, further inquiries can be directed to the corresponding authors.
